# Information Quality Challenges of Patient-Generated Data in Clinical Practice

**DOI:** 10.3389/fpubh.2017.00284

**Published:** 2017-11-01

**Authors:** Peter West, Max Van Kleek, Richard Giordano, Mark Weal, Nigel Shadbolt

**Affiliations:** ^1^Faculty of Health Sciences, University of Southampton, Southampton, United Kingdom; ^2^Department of Computer Science, University of Oxford, Oxford, United Kingdom; ^3^Web and Internet Science, Faculty of Physical Science and Engineering, University of Southampton, Southampton, United Kingdom

**Keywords:** self-tracking, quantified self, personalized medicine, information quality, health informatics, clinical decision making

## Abstract

A characteristic trend of digital health has been the dramatic increase in *patient-generated data* being presented to clinicians, which follows from the increased ubiquity of self-tracking practices by individuals, driven, in turn, by the proliferation of self-tracking tools and technologies. Such tools not only make self-tracking easier but also potentially more reliable by automating data collection, curation, and storage. While self-tracking practices themselves have been studied extensively in human–computer interaction literature, little work has yet looked at whether these patient-generated data might be able to support clinical processes, such as providing evidence for diagnoses, treatment monitoring, or postprocedure recovery, and how we can define information quality with respect to self-tracked data. In this article, we present the results of a literature review of empirical studies of self-tracking tools, in which we identify how clinicians perceive quality of information from such tools. In the studies, clinicians perceive several characteristics of information quality relating to accuracy and reliability, completeness, context, patient motivation, and representation. We discuss the issues these present in admitting self-tracked data as evidence for clinical decisions.

## 1 Introduction

Apple, Google, Microsoft, Fitbit, Withings, and Nike are among dozens of brands in the fast-growing consumer market of digital tools for keeping track of one’s daily activities, fitness, health, and wellbeing (1). The proliferation of such devices has made the activity of *self-tracking* (2)—the collection of recording data about oneself—mainstream, by enabling such recording to be done with unprecedented granularity, at little effort or cost. While the first waves of such devices have focused on apps that help individuals achieve short-term health and fitness targets, potential longer term uses for such *patient generated data* created by such self-tracking practices have been proposed. In the UK, the recent *Personalised Health and Care 2020* policy anticipated that by 2018 data generated by self-tracking products should start to become integrated with patient health records, allowing clinicians to act on the detailed data that patients record (3). This policy envisioned that patients contributing self-tracked data would be a key element toward improving healthcare quality and outcomes, while reducing costs. At the same time, patients would become empowered through use of this data, and have a better understanding of their own health. Literature on self-tracking, including those studying active participants of the *Quantified Self* self-tracking movement (4), have supported these arguments (5) and advocated for self-tracking as a way to reduce healthcare costs in monitoring chronic illness, while Swan et al. concluded that self-tracked data could serve a key role in preventative and precision medicine (6).

The *Fitbit Surge* is one such wearable self-tracking device, which automatically tracks heart rate, sleep patterns, physical activity, and distance moved. The device connects with a mobile app which displays self-tracked data as progress toward daily goals and plots over time. A substantial number of people own a Fitbit device, with 3.4 million sold in second quarter 2017 (7). Fitbit and other similar tools have made self-tracking accessible to the masses by simplifying the collection of data about health, and by presenting data in simple, easy-to-understand formats (8). Other popular products record a diverse array of information streams relating to health, such as the Apple Watch, which can record physical activity and sleep, the Caffeine Tracker app, which allows people to log how much caffeine they consume, and Daylio, an app for tracking ones mood over time.

The popularity of self-tracking has provoked interest for supporting clinical decisions, with self-tracked data “bridging the gaps” between clinical consultations and letting doctors build an accurate picture of patients over a long period of time (2, 9). These kinds of data are more diverse and detailed than traditional clinical measurements (10) and describe daily activity patterns (11) and first-hand descriptions of patient experience (12). Health tracking has been introduced into some workplaces to encourage health and wellness in employees (13). Automatic tracking through sensors, as opposed to manually recording activity, has been particularly successful at encouraging greater physical activity and health promotion (13). When collected on a population scale, data from wearable devices could provide clues to the causes of chronic conditions, such as the walkability of a city on the amount of physical activity of its citizens, which, in turn, could help shape public health policy (14).

However, several concerns have been raised about the quality of information from self-tracking practices. First, self-tracking devices have unknown reliability and validity, with most wearable device manufacturers providing no empirical evidence to demonstrate the effectiveness of their devices (15). Self-tracked data may be perceived as less reliable than medically proven lab data, and presenting self-tracked data with lab data could confuse clinicians about what data they can reliably use (16). This may be exacerbated by a high risk of patients’ use of self-tracking tools lacking scientifically rigor (17). For example, there may be a high margin of error when patients without medical training try to associate symptoms to patterns in their data (15). It has been suggested that such tools could be designed to fail in conditions where patients are trying to form unscientific conclusions (17). Second, there may be different representations of data between different patients. Patients have different preferences for self-tracking, and supporting these preferences lowers the burden for self-tracking. However, this may result in different data representations, which could create challenges for their integration into clinical care (18). Finally, the consistency of self-tracker use and abandonment may be challenges in the clinical use of self-tracked data. Users of trackers typically do not self-track on a daily basis, but take breaks every few days, and trying to force users to change this behavior will likely lead to abandonment (19). Development of frameworks for using self-tracking devices in healthcare could provoke suitable validation of data from such devices (15).

Were self-tracked data to be used in clinical decision-making, an essential threshold would have to be crossed: self-tracked data would have to be determined to be of sufficient quality to be of use in clinical decisions. In clinical settings, information quality is assured today through a multiplicity of measures, including protocols relating to methods of measurement, calibration and testing of instruments, and how the data are stored, retrieved, and used (20). In the context of patient-generated self-tracked data however, no such guarantees can be made. Self-tracking tools produce data which are new and unfamiliar to clinicians, and are seldom clinically validated (21). One study of pain management apps found that just 0.4% of apps had undergone clinical evaluation (21). How, then, can quality issues of information captured during self-tracking be resolved to make them suitable for clinical applications, such as in care delivery or personalized medicine?

This paper investigates the following research question, which we see as foundational to the clinical use of self-tracked data: *how do we understand information quality in the context of self-tracked data?* By better understanding information quality, we can more usefully forecast how such self-tracked data can support clinical uses, as well as identify the challenges that remain before greater clinical adoption can be achieved. As such, we aim to address five core questions:

- How do perceived accuracy differences in self-tracking tools affect whether and how device data are used in workflows?
- What information quality issues have been discussed previously in the use of data of digital self-tracking and paper based diaries?
- What kinds of self-tracked data were perceived as potentially the most useful by clinicians, and what were information quality needs for each?
- Are needs pertaining to information quality the same for all uses and types?
- How and where (in workflows) are issues of self-tracking compliance and motivation most salient and incorporate consideration of factors that affect compliance?

To investigate this question, we sought to understand how doctors perceive the quality of information from self-tracking practices. We conducted a literature search and review, broadly inclusive of different kinds of self-tracking (both digital and paper based), scoped only to include empirical studies pertaining to data use by clinicians and other medical professionals. We found that existing empirical research addressing self-logged information quality in clinical use was scarce, mostly limited to specific chronic care contexts, such as irritable bowel syndrome (9), pain management (22), and heart failure (23). We analyzed these studies to identify the specific information quality issues encountered, and organized these into themes.

We found a common set of challenges around accuracy and reliability, completeness, context, patient motivation, and representation. We discuss implications that these themes have for the short- and long-term use of self-logged data in clinical practice.

## 2 Materials and Methods

**Figure 1:**
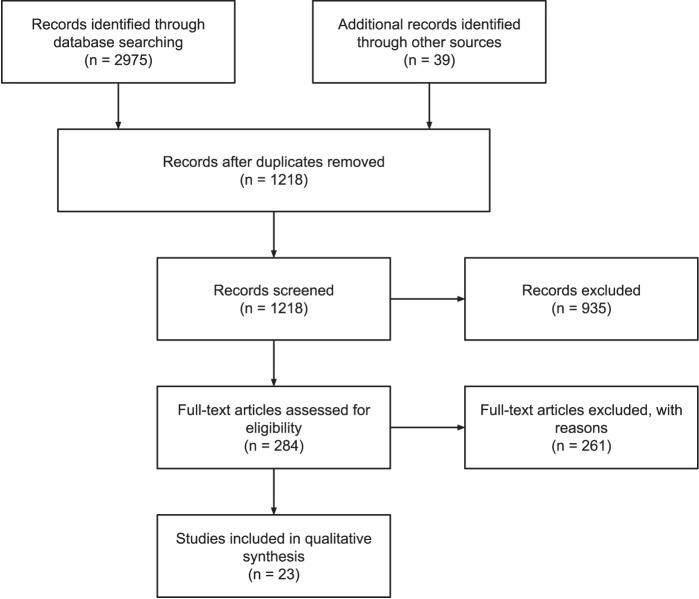
PRISMA flow diagram of the literature review.

A survey of the literature on self-tracked data was conducted to analyze the current knowledge on clinical use of self-tracked data. We use the *Preferred Reporting Items for Systematic Reviews and Meta-Analysis (PRISMA)*, which is an evidence-based minimum set of items for reporting systematic reviews or meta-analyses (24) as an audit trail of selected articles. See Figure 1 for the PRISMA flow diagram of this literature search.

### 2.1 Search Strategy

Searches were conducted on JSTOR, EBSCOhost (MEDLINE), PubMed, Cochrane, Web of Science, Scopus, and ACM DL databases (see Table 1). These databases were chosen because they index high quality journals, and encompass a multidisciplinary knowledge base. A search strategy was constructed to find literature relating to self-tracking in clinical settings:

“patient*” AND (“clinic*” OR “provider*” OR “doctor*” OR “*care professional*” OR “ambulatory”) AND (“self track*” OR “activity track*” OR “life log*” OR “lifelog*” OR “quantified self” OR “quantified patient” OR “patient generated” OR “consumer health information tech*” OR “consumer wearable*” OR “personal informatics” OR (“self monitoring” AND “diary”))

**Table 1:** Search strategy.

Database	Discipline	Records
JSTOR	Multidisciplinary	109
EBSCOhost (MEDLINE)	Medicine	366
PubMed	Medicine	504
Cochrane	Medicine	1
Web of Science	Multidisciplinary	321
Scopus	Multidisciplinary	711
ACM DL	Computing	39

Total		2,975
Total (without duplicates)		1,218

Because the phrase *self-tracked data* has many synonyms and related terms (including *life-logging, quantified self*, and *patient-generated information*), the search query was expanded to include these terms. The term *self-monitoring* generated a vast number of results relating to prescribed practices and clinical devices unrelated to self-tracking, hence *diary* was added to limit results to patients self-tracking their health. Wildcards were used in the search query to match similar words (for example, track* matches both *tracked* and *tracking*, and Boolean operators were used to constrain results to those with a sufficient set of relevant terms. All databases supported wildcards and Boolean operators, though some required the query to be split over multiple searches.

### 2.2 Selection Criteria

To focus on clinicians’ perceptions on information quality issues, we focused only on studies in which medical experts (comprising doctors, nurses, specialists, and others from primary and secondary care) accessed, used, or otherwise interacted with patient generated data (PGD). Because we were not assessing the clinical effects *per se* of self-tracking or the use self-tracked data, we did not use a standard appraisal tool, such as CASP, or a formal published checklist when selecting the papers (25). Inclusion was based on three criteria. First, the article must have been peer-reviewed, and represent empirical work, thus excluding opinion, literature surveys, and self-experimentation pieces. Second, the subject matter must have pertained to data collection by the patient using non-clinical tools. This included consumer devices, paper diaries, and patient-facing portals, but excluded telemonitoring, implantables, and other forms of data or technology used in clinical settings. Studies relating to patient-reported outcomes were also excluded, as these forms of data were usually retrospective forms of information gathering (26), rather than pervasive self-tracking. Third, the findings had to pertain to how self-tracked data were used by clinicians, including how information quality was evaluated, and used in decision-making. This excluded papers solely concerned with self-tracking for self-reflection or self-improvement. Studies about using self-tracking for research, or “big data” uses, and reviews of efficacy of self-tracking techniques were excluded as these did not relate to a clinician’s perceptions of information quality.

Several papers which were not present in the search results but were known to the authors were also included. In total, twenty-three papers met the inclusion criteria. Of the papers excluded, 996 did not pertain to patient self-tracking, 129 did not consider clinician’s perspective, 55 were not empirical, 9 were not peer-reviewed articles, 4 concerned big data only, and 4 were studies similar to other papers in the selection.

### 2.3 Analysis

Papers were divided and read through by three members of the research team to identify and tabulate clinical setting, study rationale, methods, and key conclusions from each article on self-tracking in clinical settings. Three members of the team derived themes related to information quality based on the conclusions of the articles. These themes were refined by all team members.

## 3 Results

Twenty-three papers resulted from our literature search; these are summarized in Table 2. Within the literature, we identified six themes relating to information quality of self-tracked data, which are listed in Table 3 with the papers they were identified within. In this section, we describe each of the six themes in order of their prominence within the literature.

**Table 2:** Empirical studies in clinical settings, listing the overview, method, and findings pertaining to information quality of each study.

Reference	Study overview	Method	Findings
(27, 28)	Clinicians often lack complete or accurate information about patients with multiple chronic conditions (MCC) leading to poor care coordination and medical errors	Interviews 7 MCC clinicians and 22 MCC patients about self-tracking of health-related information (e.g., test results, medications) using paper or electronic tools	Problems arise from difficulty of self-tracked data retrieval, perceived emotional valence of data, concern that the patient seems obsessive, and concern that data ma be selective reported (e.g., to avoid insurance premiums). Clinicians pursue clinical measurements over self-tracked data, though data of any form is preferable to none; patient information allows best decisions over care
(29)	Migraine often undertreated due to impaired clinician–patient communication and clinicians underestimate migraine severity	Questionnaire of 118 patients and 22 clinicians about clinician-initiated self-tracking using paper diary with questionnaires about pain, disability, and medication	Self-tracked data improves patient-clinician communication and increases patient satisfaction, possibly due to more time being spent with patient. It enables assessment of pain intensity and disability and subsequent prescription of medications
(30)	Management of weight loss is difficult, leading to underdiagnosis and undertreatment	Analysis of 30 patients following 1 month use of a clinician-initiated self-tracking app and wearable sensors	Only a few participants shared data with doctors, despite this being a feature of the app. App usage negatively impacted patient–doctor relationship
(9)	Patients with irritable bowel syndrome (IBS) often self-track lifestyle changes are often dissatisfied with feedback from clinicians despite its potential to improve efficacy of behavior change efforts	Twenty-one clinicians who work with IBS patients interviewed about their current and potential uses of patient-initiated self-tracked information from paper or electronic tools	Self-tracking tools lack flexibility and standardized formats, and doctors have a lack of time and skills to interpret such data. Nonetheless, it helps understand IBS patients’ routines and enhances clinician–patient communication and personalization of treatment plans. Contextual information helps better understand the patient. For, diagnosis high reliability and granularity is required
(31)	Builds on (9)	Surveys of 211 and interviews of 18 IBS patients, and reanalysis of 21 clinician interviews from Ref. (9) about self-tracking of health-related information using paper or electronic tools	For collaboration with patient, clinicians create “compilation artifacts” from self-tracked data by collecting information from different sources, organizing it, and presenting it. Some clinicians prefer paper for better interaction affordances. It is important to highlight missing data or mistakes for accountability
(32)	Builds on (9)	Interviews of 10 IBS patients and 10 clinicians who work with IBS patients. Clinician-initiated self-tracking of food and symptoms using bespoke mobile app	Doctors wanted to know how patient data compares to population-level data. Clinicians trust patients to interpret their own data during visits to support communication. Contextual information is important for trust, though doctors lacked trust in patient’s judgment of symptom severity. Data can support hypothesis generation on what may be causing a patient’s symptoms
(33)	Support self-management for healthy eating and preventing and managing disease	Interviews of 13 study designers and 12 healthcare professionals. Self-initiated and clinician-initiated self-tracking through a range of apps, sensors, and websites	Self-tracked data provides a deep and accurate insight into patient’s condition between clinic visits
(23)	Rehospitalization is a common occurrence for patients with heart failure (HF); preventing rehospitalization could reduce costs and decrease mortality	Observation of 124 HF patients using a diary for 6 months and analysis of clinical outcomes. Clinician-initiated self-tracking using paper or computerized diary for recording weight, symptoms, and any other behavior patients considered relevant	Diary-taking encourages more frequent contact with clinicians. Early reporting of health changes can strengthen program effect
(34)	Self-management using a Fitbit could encourage lifestyle changes which prevent chronic disease	Seventeen people at risk of chronic disease from obesity completed a study of self-monitoring using fitness trackers and perceived quality of life. Mandated self-tracking using fitness trackers and patient surveys	Self-tracking shifts responsibility of health to the patient and helps alleviate time constraints of primary care physicians
(35)	Patients not only want access to various medical records their health care providers keep about them but also are willing to become active participants in managing their health information Personal health records (PHRs) were developed to help fulfill this need. There is little understanding of different health care practitioners’ views of PHRs, including how PHRs could fit into the existing health care system, is lacking	Twenty-one clinical practitioners with 10 different specialties were interviewed. Semi-structured interviews where participants were asked to describe an “ideal” personal health record	History must be presented chronologically. Different types of information are more important to some clinicians (e.g., Chinese medicine) than others. Charts and time lines are critical
(36)	Breast cancer patients often have difficulty managing their own health information due to changes in health and goals over time	Interviews of 12 breast cancer patients. Clinician-initiated self-tracking using tablet with Note taker, MyFitnessPal, and other cancer apps	Self-tracking supports communication with doctors because patients feel more prepared and confident; patient feels empowered. Mobility of the tool allows patients to readily retrieve information and questions for the doctor
(37)	Self-tracking may help in prevention and management of lifestyle diseases	Observation of 20 lifestyle disease patients and 6 specialists with self-tracked food logs, steps, and sleeping time from a mobile app	Self-tracking enables more thorough history taking. Raw data required for additional analysis, and visualizations or summaries for “at a glance.” Clinicians expect low adherence rate and don’t have confidence in the accuracy of self-tracked data. Clinicians overlap self-tracked data with other data (e.g., sleep onto calorie intake). Although this isn’t usually enough evidence to determine cause and effect, it helps understand the patient by showing context
(22)	People with chronic illness may be able to better self-manage using self-tracking	Interviews of 12 chronic illness patients who self-track. Self-initiated self-tracking using apps and paper to track episodes, triggers, medication, status, and history	Self-tracked data facilitates effective doctor–patient communication and identification of immediate specific needs and triggers of episodes. For poorly understood conditions, self-tracked data may be the only factor for deciding how to manage their condition
(38)	Hospitalized patients may have a better experience and improved health outcomes if they are treated as stakeholders in their care and if clinicians have better access to health information	Twenty-eight inpatients and their caregivers were interviewed and observed at bedside to understand their interactions with clinicians. Self-initiated self-tracking of various forms	Self-tracking may prevent medical errors by providing information when the clinician doesn’t have it
(39)	Mobile health and patient-generated health data are promising health IT tools for delivering self-management support in diabetes, but little is known about provider perspectives on how best to integrate these programs into routine care. Provider perceptions of a patient-generated health data report from a text-message-based diabetes self-management program are explored	Twelve primary care physicians and endocrinologists. Individual interviews and survey	Respondents reported that data were more reliable from a smartphone than from a clinic visit because patents were less willing to “please the doctor.” Data were useful in understanding the patient, developing a focus in a treatment plan, increasing patient engagement, and understanding the patient’s perspective. Self-generated data did not directly influence care, but instead enhanced it
(40)	Breast cancer patients often have difficulty communicating symptoms to clinicians	Twenty-five breast cancer patients were interviewed about self-tracking and observed at home and in clinical visits. Self-initiated self-tracking of symptoms using bespoke mobile app or patient’s own technique	Self-tracking supports communication with clinicians by helping patient’s recall events and providing a basis for prioritizing symptoms. Support patient reflection and doctor–patient communication by allowing overlapping of graphs to see co-occurrences of symptoms and events. Enable clinicians to explain salient parts of the data to patients so they can understand what is happening
(41)	This paper discusses mediation in the patient–provider relationship arising from the introduction of digital technology for a specific form of monitoring: “clinical self-tracking”	Twenty-one diabetes 1 patients; 4 doctors, 8 parents, and 2 nurses at three clinics in Italy. A smartphone application enabled patients to keep track of all the information relative to their diabetes; web-based dashboard accessible by doctors with a system of rule-based alarms designed to send an alert to clinicians and/or patients in the presence of certain data or combinations of data, and a messaging platform that worked as a secure email service between patients was developed and trialed over 3 months. Interviews with respondents to evaluate it	Adolescents and adults with poorly controlled diabetes did not share data with providers; the level of autonomy is different based on patient type (e.g., children, pregnant women). Intended users of the system are not presented with a binary choice between use and non-use; rather, they enact technology selectively to fit into their lives. Authors suggest that “pushing” self-tracking by the clinician might be perceived by patients as being intrusive
(46)	Paper food and gastrointestinal symptom journals used to help irritable bowel syndrome (IBS) patients determine potential trigger foods. The study evaluated the feasibility, usability, and clinical utility of such journals as a data collection tool, and explore a method for analyzing journal data to describe patterns of diet and symptoms	Caucasian males (N = 13) women (N = 14) Mean age 35 ± 12. Participants logged three sets of 3-day food and symptom paper-based journals over a 15-day period. Subjects participated in follow-up interviews	Over half perceived paper journaling of food and symptoms as feasible, usable, and clinically useful. Thirteen participants demonstrated a strong association with at least one symptom and meal nutrient. No mechanism to capture time of completion or accuracy of entries. Journal entries for IBS patients are shown to be feasible, usable, and have clinical utility. Paper-based entries have weaknesses related to accuracy and veracity since there are no automatic mechanisms to check for these. IBS patients are possibly more motivated than others to complete entries
(42)	Consequences of personal health records on the health care system are poorly understood, in particular the temporal and cognitive burden associated with new workflows that include PGD. This paper reports the results for time-cost and resource utilization of a “typical” ambulatory clinic under varying conditions of PGD burden	The time-cost impact of patient-generated data is measured using discrete event model (DEM) simulation. Three simulation scenarios of everincreasing PGD impact are compared to a baseline case of no PGD use. A simulation with doctor, nurse, support staff roles and their costs. Real clinicians were used to estimate time, and to generate a range of assumptions that encompassed their estimates. The model was validated by subject matter experts who found that the simulator behavior they observed was consistent with clinic operation as they were accustomed to it	There are close ties between PGD use and resource utilization, including clinic layout and workflow design. Lengths of both workday and patient visit were extended and less predictable with PGD use. The integration of PGD in clinical work flows needs extensive preparation because the impact of PGD use is non-trivial and will quantifiably either cause longer workdays or mandate the sacrifice of other activity to reap any argued or measureable benefit
(44)	Decisions in diagnostic settings must often be made with very little information. Self-tracked data may be useful as evidence in diagnoses	Ten primary and secondary care physicians from UK and USA role-played two diagnosis scenarios relating to migraines and heart conditions. While participants saw potential in self-tracked data being used as evidence, in practice data were considered untrustworthy due to unknown patient motivations and unclear reliability of the recording device and technique	Self-tracked data in diagnostic settings may be dismissed as unreliable or untrustworthy. A patient’s motivation for self-tracking, the routine they followed to take measurements, and the device they used influence a doctors decision to use the data
(43)	Long-term measurements in a home environment could support screening and medical treatment. This paper describes considerations and recommendations for the design of sleep monitoring tools	Observations of 8 staff at two clinical sleep centers in Belgium, and 1 ambulatory sleep center in the Netherlands while preparing, executing, and processing sleep studies. Notes were translated to an affinity diagram that was used to uncover patterns in the interviews and observations. No community self-tracking; polysomnography in clinical settings	Doctors want the raw data to analyze for themselves. Self-monitoring sleep tools should fit in existing hardware and software, using existing standards. Sleep clinicians depend, in part, on video when monitoring sleep. Patient collected data need to contain context that substitutes for video
(45)	Patients are increasingly tracking and generating an increasingly large volume of personal health data from wearable sensing and mobile health (mHealth) apps, and that the potential usefulness of these data is enormous. This study explores how patients and clinicians currently share patient-generated data in clinical care practice	Twenty-one participants (12 patient participants and 9 clinician participants). None directly. Sample population used both clinically directed and self-directed tracking. Results derived from telephone, Skype, and face-to-face semistructured interviews from a purposive sample of patients and clinicians. Interviews were coded by two researchers	Doctor directed self-tracking motivated by desires to increase patient engagement; assessing a condition over time; ascertain the effect of lifestyle changes on health; belief that clinician authority motivates patients to track. Clinicians cannot effectively use patient generated data until it is integrated into the clinical systems. Clinicians need to be incentivized to incorporate patient generated into their everyday work flows. Clinicians need to adapt to the cultural shift in healthcare, in which more patients are attempting to make healthcare a collaborative endeavor

**Table 3:** Themes identified within the literature review.

	(27)	(28)	(29)	(30)	(9)	(31)	(32)	(33)	(23)	(34)	(35)	(36)	(37)	(22)	(38)	(39)	(40)	(41)	(42)	(43)	(44)	(45)	(46)
Structure and presentation																							
Trustworthiness and use as evidence																							
Completeness and selective memory																							
Patient motivation																							
Reliability																							
Context																							

### 3.1 Structure and Presentation

The most prominent theme deriving from our analysis pertained to the ways that self-tracked data were structured and represented. Clinical information systems, including EMRs, as well as diagnostic devices and systems, represent patient data in largely standardized ways. Such standardized representations help clinicians efficiently and accurately interpret data (9), even when such data are multivariate and voluminous. Self-tracking tools, however, were found to rarely, if ever, use or derive representations based on these standards. Several reasons have been posited for this disparity; one, for example, that most self-tracking tools are developed by tech companies with little expertise in clinical informatics. Moreover, these apps are typically created for consumers, and not for clinical purposes. Other plausible reasons are that these apps are deliberately designed to be simple, easy-to-use, and to avoid representations that could be perceived as potentially too technical in order to appeal to non-specialist individuals (9).

Beyond being unlike clinical representations, there appears to be significant variation across different self-tracking tools, whether wearable sensors (30, 33, 34), or electronic self-tracking apps (9, 22, 23, 31–33, 36, 37, 41, 47). Such variations extended beyond the specific visual representations used to present and summarize the data, to variations of data granularity, aggregation methods, to units of measure. When patients used more traditional methods, such as keeping hand-written notes, or using general-purpose tools such as word processors or spreadsheet tools, the result was often similarly varied but for different reasons; patients were seen to naturally structure their data in ways most intuitive to them, which was often idiosyncratic to their preferences and goals. Both kinds of variations and disparities of representation were seen as a direct and primary obstacle to quick, safe, and effective use of self-tracked data.

The relative importance of the use of specific representations also depended on the role of the expert reviewing the data. Primary care physicians and nurses were often more flexible in terms of “piecing together” disparate evidence, based on heterogeneous, varied information sources, including data from self-tracked tools and recounted personal experience (44). Secondary care specialists, however, more often expressed the need to reorganize, reorder, and sometimes restructure information into standardized forms (such as the clinical admissions form) before being able to effectively evaluate it (44).

The nature and kinds of relationships sought in the data often shaped the representations that were seen as most helpful. In one study, clinicians said that temporal relationships were the most important, as they established causal relationships between potential triggers and symptoms. As a result, timelines were seen as the most natural way to represent patient self-tracked diaries (35). In such timelines, both ordering (indicating precedence) and temporal distance were viewed as important for suggesting or ruling out causal links; for example, a symptom occurring before, or too long after a potential cause would suggest the two unrelated, and a timeline would make such connections quickly identifiable (44).

Typically, clinicians were unclear on how self-tracked data could be effectively presented, primarily because the data describe unfamiliar forms of measurement, such as sensor data from an accelerate. Clinicians are responding to self-tracking practices as new forms of data collection that are unfamiliar. However, as these practices and tools evolve, it is likely that the perspectives of clinicians will change, and an understanding of the next set of challenges for presenting information will become clear.

### 3.2 Trustworthiness

The term *trustworthiness* is often used in qualitative analysis (48) to describe the degree to which one can “gain knowledge and understanding of the nature of the phenomenon under study” (49). This is preferred to terms such as *accuracy* or *reliability* which describe a specific instrument’s ability to measure the phenomenon (50). These aspects of trustworthiness were often perceived by clinicians as an aspect of information quality. We appropriate the term to reflect the broad set of concerns expressed toward gaining an effective understanding of the patient through their self-tracked data. This definition is left intentionally broad to be able to accommodate the particular concerns relating to distinct uses reported across the literature, ranging from recollection of symptoms and potential triggers (23, 29, 32, 47), to helping clinicians identify long-term trends (22, 23, 43), to understanding symptomatic burden and impact on patients’ overall quality of life (36).

For differential diagnosis, trustworthiness often corresponded to quantitative measures of data quality. This included accuracy, such as whether the devices and sensors used by patients to self-measure were accurate (51), as well as reliability, such as for self-report measures in health diary apps. For the former, considerations included the type of devices used take measurements, whether the devices were clinically calibrated, and whether they used sensing approaches analogous to those used by clinical instruments (31). Other concerns included issues of data sampling, including representativeness, and completeness of data, which depended on such as issues whether the data were recorded automatically or manually, and patients’ ability and willingness to do so (45). Such factors that influenced trustworthiness included the duration over which data were logged (35), as well as granularity (9). When patient compliance with data collection was sometimes poor, resulting in gaps or insufficient detail, these were seen as negatively impacting perceived trustworthiness (37). Moreover, clinicians feared that poor compliance was potentially indicative of selective reporting, which was seen as a means of concealing information from the clinician (27).

Across the literature surveyed, self-tracked data were, in general, considered less trustworthy than corresponding clinical data. This affected how self-tracked data were treated when used at various stages within workflows. For example, in differential diagnosis, prognostic decisions based on self-tracked data were made only after considering substantial additional supporting evidence, which was systematically sought (27). The most common approach to obtain more trustworthy evidence was running additional clinical diagnostics, which was done routinely to support hypotheses whenever it was practical (27, 44).

It is important to note, however, that trustworthiness was not always an important consideration. For example, at the point in a diagnostic process that all working hypotheses are ruled out, the opportunity arises to re-consider all available information toward new hypothesis formation (44). For this purpose, it was perceived valuable to consider all available information as potential evidence, regardless of perceived trustworthiness (27). In addition, when data were examined cooperatively with patients, such as for reflection (31) or to facilitate self-recall (38, 40), notions of trustworthiness of the data were less important than the personal significance and communicative roles served by the data. For such uses, instead of seeing self-tracked data as an extension of clinical data, e.g., as an evidential basis upon which to make clinical decisions, are more appropriately thought of as “boundary objects” through which the patient and clinician can achieve a shared understanding of the patient’s condition (32).

### 3.3 Patient Motivations

Understanding the patient’s motivations has been long discussed as a key challenge in effectively addressing patient concerns and delivering appropriate care (52, 53). The motivations of the patient, and therefore the patient’s behaviors toward data collection, were perceived by clinicians to be an aspect of data quality. In the context of patient-generated data, understanding the patient’s reasons for (as well as for not) self-tracking were sometimes considered in the process of understanding the patient’s condition. For instance, the act of avid self-logging was sometimes seen as an indicator of obsession, compulsiveness, or significant concern about particular symptoms (28, 44, 45). On the other hand, a lack of self-logging compliance, or ability to keep track of symptoms was seen as potential signs of apathy (54).

Sometimes, the act of presenting data to clinicians was seen to indicate some explicit underlying motivation, such as the desire to confirm a diagnosis, or worse, “begging for a diagnosis.” Sometimes, the presentation of information was seen as a workaround for the inability to communicate something difficult to put into words. Usually, however, the presentation of PGD was simply an artifact of the fact that self-tracking was on the rise, and that patients felt such data would be useful for clinicians during the consultation (9, 44).

In the context of long-term health management, the motivations for self-tracking usually were unambiguous, relating to chronicling of symptom burden and other issues of concern to them (9, 23, 37). When managing multiple chronic conditions, however, self-logging was often not perceived to be a reliable indicator because it was seen as both an excessive burden for patients who had already experienced significant disease burdens that limited their daily activities (27). Moreover, the lack of an evidence base for combinations of symptoms those with multiple chronic conditions, meant that logs for these patients were more ambiguous to interpret, and more difficult to translate to effective outcomes, and thus were seen as less of direct value to the patient.

### 3.4 Capture Context and Metadata

Contextual aspects of how self-tracked data are captured, such as location, time of day, activity, and posture, are important for understanding cause and effect where multiple medical conditions exist (43) and incomplete information contributes to medical errors (27). Single data streams on their own may provide insufficient context about a patient’s recordings for clinicians to make suitable judgments from them. Self-tracking devices today rarely capture such contextual dimensions, and typically only capture limited metadata about measurements (55); self-tracking tools tend to focus on a single facet of people’s lives (56) and users are not aware of important information that they do not collect (57). However, self-tracking devices, such as wearable sensors, provide a means of capturing data at high-resolution and granularity with little or no effort to end-users, supporting creation of time series datasets with a high degree of completeness (9). Future technology may be capable to automatically collating information from separate data sources to provide contextual information about a patient’s recordings.

### 3.5 Format and Representation

The choice of format affects the clinician’s ability both to interpret the data and trust it (32). The availability of contextual information (such as what the patient was doing and where they were during a measurement) is also a factor in trust (32) and contributes a better understanding of the patient (9). Contextual information could consist of other data sources available in a patient’s health record or through self-tracking other kinds of information (32). Overlaying population-level data onto data visualizations may help in interpreting types of measurement uncommon in clinical settings (32, 40).

Healthcare information systems often do not provide ways to interoperate with self-tracked data (9, 27, 37), and data which is entered into these systems are prone to getting lost (38). Thus, doctors are pragmatic in how data can be retrieved, with any data being better than none (27). Woods et al. (58) note that clinicians are more likely to accept types of data that fit a biomedical model. Similarly in constructing a risk model of integrating clinical and Quantified Self data, Third et al. (59) noted the need to adopt commonly used standards to aid integration of the data.

## 4 Discussion

In this section, we bring together the themes identified in our literature review to address the question posed in the introduction: *how do we understand information quality, in the context of self-tracked data?*

### 4.1 Synthesis: Dimensions of Self-Tracked Information Quality

**Figure 2:**
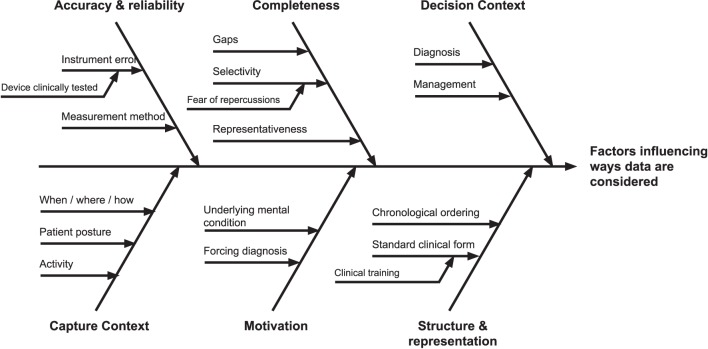
Factors influencing self-tracked data use. Toward the left of the figure are issues pertaining to data creation, through to data use and application to the decision-making context at the right.

Information quality issues were seen to affect the ways self-tracked data were considered by clinicians during a consultation in many ways. Drawing on the themes from the literature review, we synthesized a view of these factors, visible in Figure 2, as a journey from the patient’s initial collection of data, to use in clinical settings.

The most prominent theme primarily derived from our analysis was the importance of the **decision context** when interpreting important quality issues in self-tracked data. In particular, a context of differential diagnosis presented different quality needs and considerations for self-tracked data than the context of chronic condition self-management. For diagnosis, self-tracked could be used as supporting evidence in the service of deriving an appropriate plan of care, and thus establishing its veracity, provenance, and reflection of actual patient experience was viewed as crucial. For chronic condition management, however, literature suggests self-tracked data as primarily indicative of symptomatic burden and subjective patient experience, including quality of life.

A second prominent theme pertained to how self-tracked data were **presented**. It was clear from the literature that clinicians preferred patient data to be presented in particular ways, such as events ordered chronologically, or organized according to a familiar structure such as the ubiquitous hospital admissions record. It was found that patient-facing apps rarely provided presentation capabilities (such as statistical interpretation aids) on a par with those of clinical systems, because they were assumed to be used by non-specialists. As a result, underlying data quality issues aside self-tracking systems today were viewed as more time-consuming and difficult to interpret, merely from a lack of presentational support for effective interrogation.

On the far left of Figure 2 are information quality issues introduced at the creation of self-tracked data. This includes low-level information quality issues identified in the literature review pertaining to individual measurements, including **accuracy and reliability**, stemming from causes such as instrumentation error and incorrect methods of capture. Understanding how the measurements were taken (which were thematically described as **capture context**) including the patient’s posture, activity immediately prior to, and when, where, and how data were taken, were discussed as a potential mechanism of enabling the assessment of reliability of self-made measurements, in the absence of controls for how they were taken.

Beyond individual measurements were quality issues of aggregated collections of self-tracked data, including longitudinal time-series measurements. For such collections, issues of data **completeness**, and sampling arose, including interpretations of gaps in important data, and representativeness of collected data. Understanding the latent causes were discussed for each of these, including, most significantly, underlying **patient motivations** in both self-tracking and presentation. It was deemed that such motivations, including “begging for a diagnosis,” or wanting confirmation of a disease were important to consider as they were seen to naturally shape (and potentially bias) the data that patients would be inclined to present and emphasize. On the other hand, absence of data could be an indicate a lack of motivation or a lack of need to self-track, which could be interpreted either as being well or having other priorities.

### 4.2 Applying the Factors: Scenario Walk-Through

The results from the literature review now affords us the opportunity to construct a walk-through that delineates how the quality of self-tracked data, and the context of decision-making, affect the trustworthiness of information presented to a clinician. The purpose of this fictional multipart scenario, which is not based on real cases by medical experts, is purely to illustrate how the characteristics of information quality discussed in the previous section (see Figure 2) might manifest at various stages of a patient’s clinical workflow.

Let us imagine that we have a patient, Rupert, who suffers chest pains when he climbs a flight of stairs. Rupert then records his blood pressure and heart rate and brings this information to his doctor. Working from the left-hand side of Figure 2, to the right, we will uncover the story of how and to what extent Rupert’s data are trusted and used by the doctor. Given our model, Rupert’s doctor will be concerned with the context by which Rupert captured the data, how accurate and complete the readings are, and what motivated Rupert to collect those readings. The doctor will also want to see the readings in a format that is understandable and usable. Is Rupert looking for a diagnosis of his symptoms, or is he visiting the clinic as part of a pathway to evaluate and assess a treatment plan?

#### 4.2.1 Diagnosing His Condition

When Rupert presents his blood pressure and heart rate readings to his doctor, one of the first questions that will concern the doctor is how were the data collected? What was Rupert’s posture when he took his readings? How long after climbing the stairs did Rupert take his measurement? Rupert might not remember this accurately; would the doctor simply take Rupert’s word? This raises some questions in the doctor’s mind about the context in which the readings were collected; consequently, the data is suspect.

Which instrument did Rupert use? How does Rupert’s doctor know if this instrument is accurate? The doctor has a reading, but is this complete? Is it just a single reading? Is it an average of three readings? If it is an average, how much time passed between readings? Did Rupert use the device correctly?

How complete is this data to make a diagnosis? How can a simple blood pressure/heart rate reading diagnose heart disease? What gaps are there that need to be addressed to support a diagnostic judgment? Does Rupert have angina? Has he suffered a mild heart attack? Is it an esophageal spasm?

The doctor may then ask if Rupert is suffering from stress, and both the chest pains along with the data collection are merely symptoms of this.

Finally, is the data presented in a way that Rupert’s doctor can understand—are they presented in a standard format? Is it a graph? Do the readings show actual values or just a graphical rendition of them?

If Rupert expected that the doctor would use his data to support a diagnosis, he would be disappointed because Rupert’s doctor cannot trust this data to support a diagnosis. Instead, the doctor may glance at Rupert’s data out of politeness, set it aside, and decide on which tests he would order to support a diagnosis, or refer Rupert to a cardiologist.

#### 4.2.2 Managing His Condition

In this scenario, let us assume that Rupert has already been diagnosed with angina, has been prescribed medication, and his doctor is evaluating a treatment plan. Part of that plan is to visit the clinic with weekly blood pressure readings, heart rates, and activity logs. In this scenario, Rupert’s blood pressure device has been calibrated with a device in the doctor’s office, and Rupert is wearing a wearable device recommended by his doctor that logs activity and measures his heart rate. Rupert has been instructed on when and how to measure his blood pressure.

The doctor is satisfied with the context of data capture, the accuracy of the readings, and the completeness of the data. Rupert’s doctor is also satisfied that Rupert is not a member of the “worried well,” but someone who desires to manage his heart condition. The devices that the doctor has instructed Rupert to use display information in a manner that is useful to the doctor, and, as a bonus, can be transferred to Rupert’s electronic medical record. The data, while still not complete enough to support a diagnosis, is of high enough quality to document Rupert’s condition between clinic visits, and to measure the effects of a treatment plan. If Rupert complains that he has chest pains while climbing a flight of stairs, the doctor can either change Rupert’s heart medication or increase the dosage, and then ask Rupert to continue to self-collect data and visit the clinic again sometime in the future.

#### 4.2.3 Comparison of Situations

In the two above scenarios, the essential presenting complaint was the same: that Rupert experienced angina after climbing a flight of stairs. Yet the decision contexts of each dramatically shaped the needs and perceptions of self-logged data in each. In the first scenario, a lack of pre-existing condition meant that the doctor started with a much larger hypothesis space, and each subsequent decision was more critical, as it could potentially affect the patient’s ultimate prognosis. As a result, one would expect evidence, including self-tracked data, to be heavily scrutinized along the various dimensions in Figure 2. In the latter, the angina was viewed in the context of monitoring his already known condition. As a result, he might expect such data to be viewed in aggregate as part of larger trend, for informing his course of further treatment.

### 4.3 Limitations of the Study

The selected articles generally reflect the characteristics of research on self-tracked data in clinical settings. Although all of the selected articles were empirical and involved human subjects, they varied widely in sampling frames, population characteristics, study designs, and analytical techniques. Consequently, a meta-analysis from the selected work was not possible. Prior work in this area suggests that clinical studies of self-tracking are scarce (2, 60). For example, a review of pain management apps revealed that only 0.4% of apps had been evaluated for effectiveness (21). Furthermore, although there is a plethora of *Quantified Self* studies of self-tracking for health purposes, they have been predominantly small in scale (often with just a single participant), not peer-reviewed, and pertained to self-experimentation without clinical oversight (6). Moreover, studies involving self-tracking tools are often not representative of the general population, with bias in participant selection toward computer-literate people in affluent areas (27). The general population of patients is often neither computer-literate nor affluent; older patients, for example, with chronic conditions are likely to be less able and less computer-literate [(2), p. 174], despite being a population group who would be most advantaged by the personalized medicine which self-tracking could enable (28).

A second limitation of this research is that our insights, while suggestive, are not sufficient to support any particular interventions, in terms of shaping clinical workflows to support self-tracking tools, or the design of patient self-tracking tools. This highlights the importance of further empirical work and design research.

### 4.4 Future Work

The findings from this literature review provide an early overview of the challenges of using self-tracked data in clinical settings. The perspectives of clinicians from the included studies reflect that self-tracked data is a new and unfamiliar source of information. Clinicians perceived several broad themes of information quality as pertinent in deciding if and how such data could be used in decision-making. The lack of evidence from clinical trials pertaining to self-tracking tools was a significant factor in clinicians’ concerns about the reliability and presentation of such data. We believe that this highlights an important area for future work: empirical studies of self-tracked data in clinical scenarios will be increasingly important, as more patients choose to engage in self-tracking practices, as these practices will inevitably manifest themselves in clinical settings.

There is the potential that self-tracking will provide new forms of detailed information useful for a future digitally enabled healthcare. Such information could improve patient outcomes, fill the gaps between consultations, and help manage long-term conditions; these uses of self-tracked data may be critical in a time where more people are suffering from chronic illness and healthcare costs are rising (6). However, this is a new research space with only limited understanding of the opportunities and challenges of self-tracked data in clinical settings. We envision that future clinical trials of self-tracking tools and practices will uncover a deeper understanding of clinicians’ perceptions of quality of these forms of information. We see this as an iterative process by which clinicians (and researchers) will incrementally achieve a deeper understanding of all of the ultimate challenges to using data from self-tracking tools and practices.

## 5 Conclusion

In this paper, we sought to identify and understand information quality needs as they relate to the clinical use of self-logged, self-tracked data. Through a literature review, we identified issues relating to accuracy and reliability, completeness, context, patient motivation, and representation. Rather than being absolutes, however, we found strong indicators to suggest that information quality needs of such data are highly dependent on the decision contexts in which data are used. In diagnosis, data are interpreted as evidence in service of particular hypotheses. Therefore, carefully interrogating self-logged data along relevant dimensions, including for individual measurements, is important to ensure proper conclusions and appropriate diagnoses are made. However, in the context of chronic care management, the data are used essentially to support reporting. This context is simpler, which means the space of potential misinterpretations is significantly smaller. Moreover, in the former setting, the doctor and the patient have an asymmetric relationship; while in the collaborative care management context, the two parties essentially work to enhance a shared understanding of the patient’s condition and plan. In this collaborative context, some dimensions of information quality discussed, such as understanding the patient’s motivations, are self-evident and simply not necessary to consider.

In summary, we believe that is significant scope for further investigation in the use of self-tracked data in clinical practice. Self-tracking practices continue to be on the rise across individuals with many different kinds of health and well-being goals, thanks, in part, to the continuing proliferation of health and activity sensing technologies as well as apps. Thus, we feel that there is an urgent need to continue to investigate information quality issues that particular clinical uses pose, as well as potential strategies relating to clinical workflows, patient self-logging practices, or the devices themselves, toward resolving such issues. In the short term, our findings support the view that, the less challenging requirements of collaborative care management may make it a more realistic first target, rather than diagnosis, for greater adoption of self-tracked data.

## Author Contributions

Substantial contributions to the conception or design of the work; or the acquisition, analysis, or interpretation of data for the work; drafting the work or revising it critically for important intellectual content; final approval of the version to be published; agreement to be accountable for all aspects of the work in ensuring that questions related to the accuracy or integrity of any part of the work are appropriately investigated and resolved: PW, MK, RG, MW, and NS. Review response and revisions: PW, MW, and MK.

## Conflict of Interest Statement

The authors declare that the research was conducted in the absence of any commercial or financial relationships that could be construed as a potential conflict of interest.
